# Small but mighty: ATG9A-positive vesicles are a branch of the intracellular nanovesicle superfamily

**DOI:** 10.1080/27694127.2025.2513467

**Published:** 2025-06-04

**Authors:** Mary Fesenko, Stephen J. Royle

**Affiliations:** Centre for Mechanochemical Cell Biology and Warwick Biomedical Sciences, Warwick Medical School, University of Warwick, Coventry, UK

**Keywords:** Autophagy, membrane traffic, microscopy, proteomics, transmembrane protein, transport vesicle

## Abstract

The molecular and functional characterization of the thousands of uncoated intracellular transport vesicles inside cells is a major challenge. Intracellular nanovesicles (INVs) are a large and molecularly heterogenous family of uncoated transport vesicles, which are comprised of multiple subtypes. As a step to characterizing these subtypes, we recently published the first INV proteome and were intrigued by the enrichment of ATG9A in it. ATG9A is the only conserved transmembrane protein with a core function in macroautophagy/autophagy, and it is found on small, uncoated vesicles, termed “ATG9A-positive vesicles”. We therefore, set out to disambiguate the relationship between these two types of vesicular carriers in cells. We showed that ATG9A-containing vesicles, rather than being a distinct vesicle class, represent one subset of the INV family. We also demonstrated that this relationship is functionally important and that perturbing INV-mediated trafficking impeded starvation-induced autophagy. Here, we briefly introduce INVs, summarize the evidence supporting our definition of ATG9A-flavor INVs and present our outlook on why we hope that this classification will help to consolidate efforts to understand the functions of these vesicles in autophagy and beyond.

In eurkaryotic cells, proteins are trafficked between endomembrane compartments by membrane-bound vesicular carriers, into which they are selectively packaged along with signaling molecules (e.g., RAB-GTPases) and fusion machinery (SNAREs) that define the cellular destination of the vesicle. Vesicular carriers have been classically distinguished by the presence of characteristic electron-dense protein coats on their surface and the intracellular pathways on which they transfer cargos. However, the cell is filled with thousands of uncoated transport vesicles, which remain largely unexplored.

Intracellular nanovesicles (INVs) are one such class of uncoated transport vesicle. They are defined by their small size of ~35 nanometers and the presence of peripheral membrane proteins of the Tumor Protein D52-like (TPD52-like) family on their surface. Of these, TPD54/TPD52L2, has the highest expression and indeed is one of the most abundant proteins in HeLa cells, making it an excellent marker for INVs.

INVs have remarkable molecular heterogeneity. We previously showed that 16 RAB-GTPases and at least 4 R-SNAREs are found on INVs, which suggested that this vesicle family is comprised of multiple subtypes. To investigate these subtypes, we carried out a proteomic survey of INVs isolated from HeLa cells, which yielded a diverse dataset of ~600 proteins^[1]^. So where does autophagy come into the story?

The INV proteome overlapped with two other previously characterized small, uncoated intracellular vesicle types – synaptic-like microvesicles (SLMVs) and ATG9A vesicles – that had been published by the Faundez and Tooze labs, respectively. As their name suggests, SLMVs are synaptic vesicle analogs that mediate stimulus-dependent exocytosis in non-neuronal secretory cells, whereas ATG9A-positive vesicles are thought to provide the seed membrane for the phagophore and may also deliver additional lipids or protein cargoes to it through transient interactions during the entire process of autophagosome biogenesis. The presence of synaptic vesicle-associated proteins in the INV proteome made sense since we had previously shown that INVs can undergo exocytosis. However, the overlap in INV and ATG9A-positive vesicle proteomes was surprising, as our earlier work indicated that INVs do not function on the late endocytic pathway, where autophagosomes and lysosomes converge. Interestingly, the Tooze lab had found that the INV marker TPD54 was one of the most highly enriched proteins associated with ATG9A-positive membranes isolated in conditions of starvation-induced autophagy.

Given the intriguing proteomic similarities between INVs and ATG9A-positive vesicles, we sought to disambiguate their relationship in cells. To do so, we used genetic tools that allow the rapid, chemically inducible capture of vesicles at an ectopic cellular location – the mitochondria – and imaged the proteins they carry (Figure 1A). We demonstrated that ATG9A is found on INVs and, reciprocally, that TPD54 is found on ATG9A-containing vesicles. We also showed that three other ATG9A-positive vesicle proteins – SH3GLB1 (Bif-1/endophilin B1), DAGLB and PI4K2A – are also found on INVs (Figure 1B). Both ATG9A and TPD54 relocalized to the mitochondria with identical kinetics, further strengthening the evidence that they are found on the same vesicles (Figure 1C). Most importantly, while we were able to capture the entire mobile fraction of ATG9A on the mitochondria via TPD54, we could only trap ∼20% of TPD54 via ATG9A. In other words, all ATG9A-positive vesicles are INVs, but not all INVs are ATG9A-positive vesicles. Since they represent one of several subsets of the INV family, we termed them “ATG9A-flavor INVs”.

**Figure 1.**
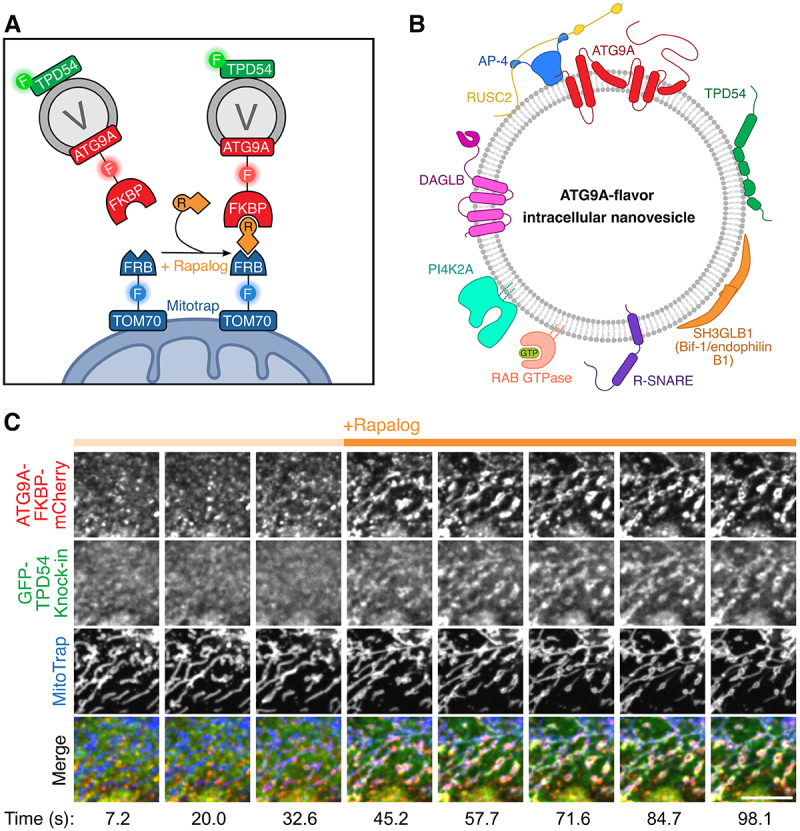
Overview of the molecular characterization of ATG9A-flavor INVs. (A) schematic diagram of the mitochondrial vesicle capture assay principle, which is based on the FKBP-FRB inducible heterodimerization system. A vesicle marker protein (e.g., ATG9A), tagged with an FKBP domain, is co-expressed in cells with MitoTrap (a mitochondrially targeted FRB domain). Addition of rapalog results in the capture of vesicles carrying ATG9A-FKBP-mCherry on the mitochondria via FKBP-rapalog-FRB heterodimer formation. Other proteins that are also on or in these vesicles (e.g., TPD54) would be co-relocated to mitochondria upon ATG9A-FKBP-mCherry relocalisation. (B) a cartoon illustrating the proteins we found to be associated with ATG9A-flavor INVs in cells. (C) cropped stills from a representative timelapse movie of the experiment depicted in (A). Scale bar, 5 µm. Reproduced from Fesenko et al. (2025) licensed under CC by 4.0.

We have also shown that INV-mediated trafficking of ATG9A is important during the starvation-induced autophagy response. Knockdown of TPD54 partially blocked the characteristic peripheral redistribution of ATG9A away from the trans-Golgi network during this process. This, in turn, could prevent ATG9A reaching phagophore assembly sites, where it is needed to facilitate phagophore expansion and could account for the lack of increase in autophagosome size and dampened autophagic flux in response to starvation in TPD54 depleted cells. These phenotypes underscore that ATG9A-flavor INVs are functionally equivalent to the previously characterized ATG9A-positive vesicles and suggest a novel autophagy-regulating role for TPD54.

One puzzling observation is that Atg9-positive vesicles were originally discovered in yeast, but *S. cerevisiae* do not have TPD54 – so, do they have INVs? We speculate that INVs evolved to meet the demands of more complex membrane trafficking networks in metazoans and, indeed, *S. cerevisiae* also does not have Adaptor Protein 4 (AP-4), another important regulator of ATG9A exit from the Golgi. Thus, yeast Atg9-positive vesicles and mammalian ATG9A-flavored INVs may be functionally equivalent despite molecular differences.

While ATG9A-positive vesicles have been best studied in the context of autophagy, there has been a growing appreciation that they are involved in many autophagy-independent processes. For example, recent work highlighted a role for ATG9A in cell migration via regulating integrin recycling, which is a process known to be mediated by INVs. Thus, we propose that rather than being considered as autophagy-specific vesicle type, the classification of ATG9A-flavor INVs as a vesicle subtype with lipid scrambling potential that can participate in a wide range of cellular activities is a more accurate definition.

## Data Availability

Data sharing is not applicable to this article as no data were created or analyzed in this study.

## Abbreviations

ATG: autophagy-related [protein]
COP-I: Coat Protein Complex I
COP-II: Coat Protein Complex II
GFP: green fluorescent protein
HeLa: Henrietta Lacks [cervical epithelial cell line]
INVs: Intracellular Nanovesicles
TPD52-like: Tumor Protein D52-like
SNARE: Soluble N-ethylmaleimide-sensitive factor attachment protein receptor
RAB GTPase: Ras-related in brain guanosine triphosphatase.
